# Hepatotoxic Mechanisms of Polyethylene Terephthalate Microplastics Revealed by Network Toxicology, Molecular Docking, and In Vivo Validation

**DOI:** 10.3390/ijms27073256

**Published:** 2026-04-03

**Authors:** Xuemei Tan, Min Zhang, Jingying Lu, Shuo Shi, Xueting Shi, Zhouhua Hou

**Affiliations:** Department of Infectious Diseases, Xiangya Hospital, Central South University, Changsha 410008, China

**Keywords:** polyethylene terephthalate microplastics, hepatotoxicity, network toxicology, PI3K-AKT signaling pathway, molecular docking

## Abstract

Polyethylene terephthalate microplastics (PET-MPs) are emerging environmental pollutants, but the molecular mechanisms underlying their hepatotoxicity remain poorly understood. Here, we combined network toxicology with experimental validation to investigate how PET-MPs induce liver injury. In silico, we investigated the PET-repeating unit as the molecular basis for target interactions. We identified 59 overlapping genes between 157 putative PET-MPs targets and 1693 liver injury-associated genes. Protein–protein interaction analysis revealed six hub genes (AKT1, PIK3CA, PIK3CB, PIK3CD, PIK3R1, and SRC), all components of the PI3K/AKT signaling pathway. Gene ontology analysis showed that PET-MPs affect cellular stress responses and kinase activities, while pathway enrichment analysis identified PI3K-Akt, Ras, and reactive oxygen species pathways as primary targets. Molecular docking demonstrated strong binding affinity between PET-MPs and these core targets (binding free energies <−5 kcal/mol). In vitro, PET-MPs induced mitochondrial depolarization, oxidative stress, upregulation of TNF-α and IL-6, and decreased p-AKT/AKT ratio, accompanied by increased apoptosis; the apoptotic effect was reversed by the AKT agonist SC79. In vivo experiments confirmed that AKT activation reduced PET-MP-induced liver injury, evidenced by decreased inflammation, lower serum transaminases, and restored oxidative balance. These protective effects were abolished by PI3K/AKT pathway inhibitors. Our study identifies potential therapeutic targets and strategies for PET-MP-induced liver injury.

## 1. Introduction

Polyethylene terephthalate (PET) is a thermoplastic polyester synthesized through the polycondensation of terephthalic acid and ethylene glycol [1]. Due to its high tensile strength, thermal stability, and chemical resistance, PET is widely used in industrial applications and consumer products [2,3]. However, long-term use, physical wear, and improper disposal cause PET to fragment into microplastics. These particles are highly stable, resistant to degradation, and readily dispersed due to their small size [2,4]. PET microplastics (PET-MPs) have been detected in the atmosphere [5], soil [6], and aquatic environments [7], creating multiple exposure routes for humans.

Microplastics can enter the human body through the skin, respiratory tract, and digestive system [8]. PET particles (50–500 µm) have been found in human stool [9], and PET-MPs account for over 50% of microplastics detected in human blood vessels [10]. Animal studies show that inhaled microplastics trigger pulmonary inflammation and impair lung cell function, with chronic exposure causing pathological changes [11]. Microplastics also disrupt gut microbiota, impair nutrient absorption, and alter systemic metabolism [12]. As foreign particles, they can activate immune cells, potentially causing immune dysregulation and increasing the risk of allergies or autoimmune diseases [13]. Because microplastics can cross biological membranes, they distribute through the bloodstream to organs such as the liver and kidney, threatening organ function [14]. Despite these findings, the toxic mechanisms and health impacts of PET microplastics remain poorly understood.

The liver performs essential functions, including metabolism, detoxification of xenobiotics, and immune regulation [15,16]. Disruption of hepatic homeostasis can cause liver injury and, in severe cases, liver failure [16]. Recent studies show that chronic PET-MP exposure causes hepatic lipid metabolism disorders and liver fibrosis in mice [17,18]. In vitro, PET-MPs promote HepG2 cell proliferation, induce oxidative stress, disrupt mitochondrial function, and activate autophagy [19]. Additionally, other studies have reported that PET-MP exposure can alter the hepatic transcriptome profile, regulate the expression of inflammatory genes, and activate signaling pathways related to liver injury, such as the p38 MAPK and NF-κB pathways [18,20,21]. However, the mechanisms of PET-MP-induced hepatotoxicity remain unclear.

Network toxicology integrates systems biology, bioinformatics, and chemoinformatics to analyze interactions between chemicals and biological systems at molecular, cellular, and organismal levels [22]. Here, we used network toxicology to investigate the hepatotoxicity of PET-MPs. We predicted the toxic properties of PET-MPs, identified key target genes, and characterized the molecular pathways involved in liver injury. Our findings clarify the health risks of PET-MP exposure and provide a scientific basis for developing interventions against PET-MP-induced liver injury.

## 2. Results

### 2.1. Evaluation of the Potential Hepatotoxicity of PET-MPs

**Figure 1:**
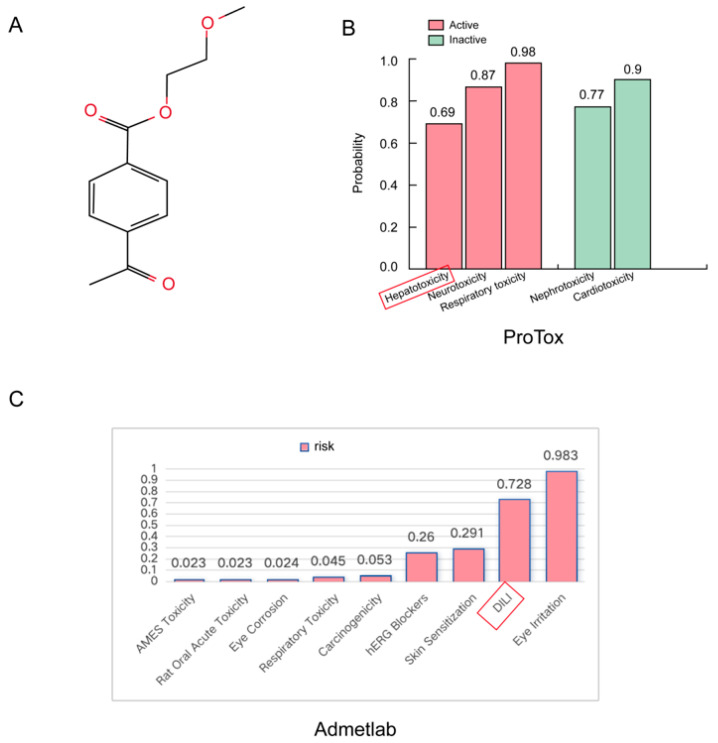
Prediction of PET-induced hepatotoxicity. (**A**) Chemical structure of the PET repeating unit used for in silico analysis. (**B**) Organ toxicity prediction by ProTox, indicating that the liver is among the primary target organs affected by PET exposure. (**C**) ADMET prediction by ADMETlab 3.0, further supporting the potential hepatotoxic risk of PET and consistent with the result of ProTox 3.0.

Due to the limitations of existing toxicity prediction tools, which are primarily designed for small molecules and cannot directly process high-molecular-weight polymers, a model compound representing the core structural unit of PET (Figure 1A) was used as the input for computational predictions. ProTox 3.0 [23], based on its large-scale compound toxicity database and structure–activity relationship models, predicted that the model compound representing the core structural unit of PET exhibits drug-induced liver injury (DILI) activity with a probability of 0.69 (Figure 1B). ADMETlab 3.0 [24] yielded a similar DILI risk probability of 0.728 (Figure 1C). These results suggest that PET-MPs have high hepatotoxic potential.

### 2.2. Screening and Determination of Hub Genes

To identify the key molecular targets of PET-MP-induced liver injury and lay a target foundation for subsequent molecular interaction network construction, hub gene identification and functional pathway enrichment analysis, this study adopted a multi-database integration strategy to screen core target genes. A total of 81 and 76 potential PET-MP target genes were predicted by the SuperPred 3.0 and SwissTargetPrediction (2019 update) databases, respectively. The union of these yielded 157 PET-MP-related target genes. For the screening of liver injury-related genes, the GeneCards, OMIM, and TTD databases provided 1651, 36, and 6 candidate genes, respectively. Merging these resulted in 1693 liver injury candidate genes (Supplementary Material S1).

**Figure 2:**
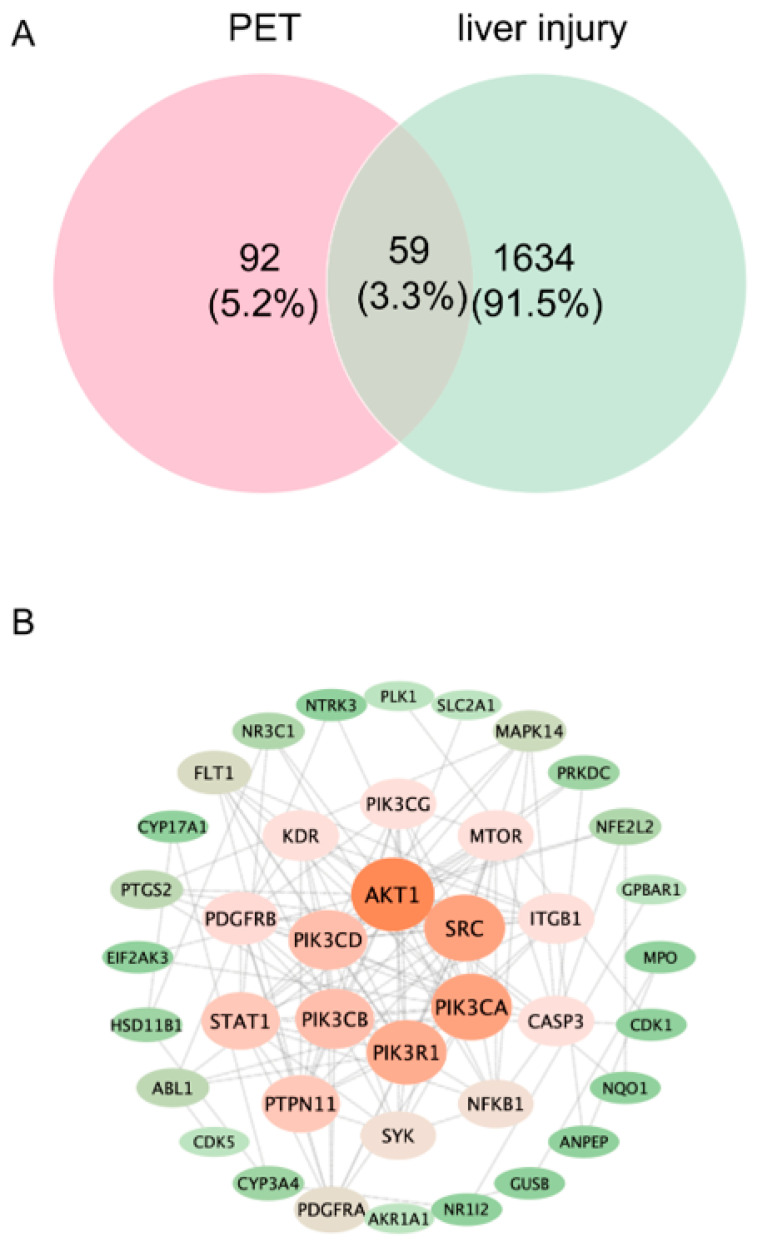
Identification of potential targets involved in PET-induced liver injury. (**A**) Venn diagram showing 59 overlapping genes shared between PET-related predicted targets and liver injury–related genes, representing candidate targets underlying PET-induced hepatotoxicity. (**B**) PPI network constructed from the top 59 key genes, in which AKT1, PIK3CA, PIK3CB, PIK3CD, PIK3R1, and SRC are identified as hub targets with high connectivity, indicating their central roles in mediating liver injury induced by PET.

Taking the intersection of PET-MP target genes and liver injury-related genes finally screened out 59 genes, identified as potential key genes in PET-induced liver injury (Figure 2A). These genes are listed in the Supplementary Material S2.

### 2.3. PPI Network Construction and Hub Gene Identification

To identify a focused set of genes that are most likely to mediate the hepatotoxic effects of PET-MPs, a PPI network was constructed by importing the 59 key genes into the STRING database with a high confidence threshold (>0.7) (Figure 2B). This network contained 40 nodes and 137 interaction edges. The network data were then imported into the Cytoscape platform for topological analysis. The top 15% of nodes, ranked by their degree of connectivity, were selected as core nodes, and a visual PPI map was constructed to preliminarily identify hub proteins within the network (Table 1).

**Figure 3:**
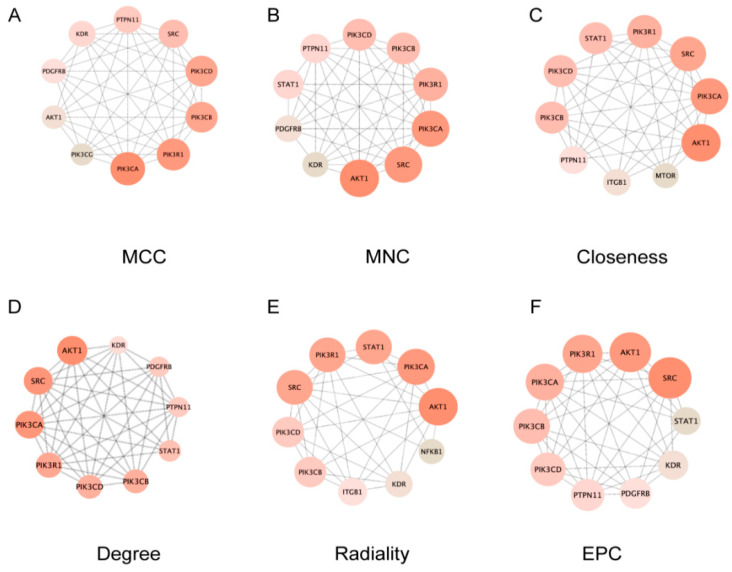
Identification of core targets using different algorithms in cytoHubba. Core target genes were identified and verified using six independent algorithms: (**A**) MCC, (**B**) MNC, (**C**) closeness, (**D**) degree, (**E**) EPC, and (**F**) radiality methods are shown.

To identify hub genes in the network, we used the CytoHubba plugin (Version 0.1) in Cytoscape with six topology-based algorithms (MCC, MNC, closeness, degree, EPC, and radiality) (Figure 3). The intersection of these six algorithms identified the hub genes as AKT1, PIK3CA, PIK3CB, PIK3CD, PIK3R1, and SRC. AKT1 ranked first in both closeness and radiality algorithms, indicating its central position in the network and its crucial role in signal propagation. In the degree and MNC algorithms, AKT1, SRC, and PIK3CA were the top three ranked proteins, suggesting they have the most extensive direct interactions. Study on microplastic-induced organ injury has confirmed activation of the PI3K/AKT pathway in this process [25], consistent with the high connectivity of AKT1 and PIK3CA observed in our analysis. SRC ranked first in the EPC algorithm, highlighting its important function in maintaining network connectivity. Previous research has demonstrated that SRC family kinases mediate hepatocyte proliferation and apoptosis during liver injury, supporting its role as a network stability node [26]. The MCC algorithm results showed that PI3K complex members (PIK3CA, PIK3R1, PIK3CD, and PIK3CB) occupied core positions, indicating that this complex forms a central functional module in PET-MP-induced liver injury. In contrast, AKT1 ranked relatively lower in the MCC algorithm, which is consistent with its role as a downstream signaling node of the PI3K complex.

### 2.4. GO Functional and KEGG Pathway Enrichment Analysis

**Figure 4:**
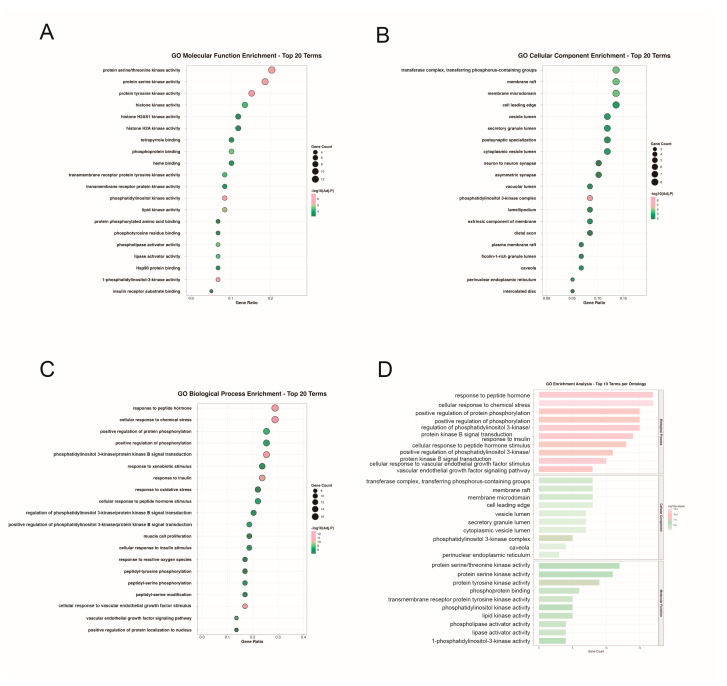
GO enrichment analysis of the 59 potential targets. (**A**–**C**) Bubble plots display the top 20 significantly enriched terms in the (**A**) biological process (BP), (**B**) cellular component (CC), and (**C**) molecular function (MF) categories, ranked by false discovery rate (FDR). (**D**) Bar graph showing the top 10 most significantly enriched terms from each GO category.

To investigate the molecular functions and signaling pathways associated with PET-MP-induced liver injury, we performed GO and KEGG enrichment analysis using the 59 candidate genes as input in the DAVID database, with the species restricted to Homo sapiens. A total of 1201 significantly enriched GO terms were identified, including 1108 biological process (BP), 33 cellular component (CC), and 60 molecular function (MF) terms (Supplementary Material S3). The top 30 terms with the lowest FDR in BP, CC, and MF were visualized (Figure 4). BP enrichment showed activation of chemical stress response and positive regulation of phosphorylation in PI3K/AKT and VEGF pathways. CC analysis revealed clustering of membrane signaling platforms (membrane rafts, caveolae) and subcellular structures (PI3K complex, perinuclear ER). MF analysis demonstrated upregulated kinase activities (protein tyrosine, serine/threonine, lipid kinases) and enhanced phosphorylated protein binding. These results suggest that PET-MPs induce liver injury through disruption of stress response, signal transduction, and metabolic homeostasis.

**Figure 5:**
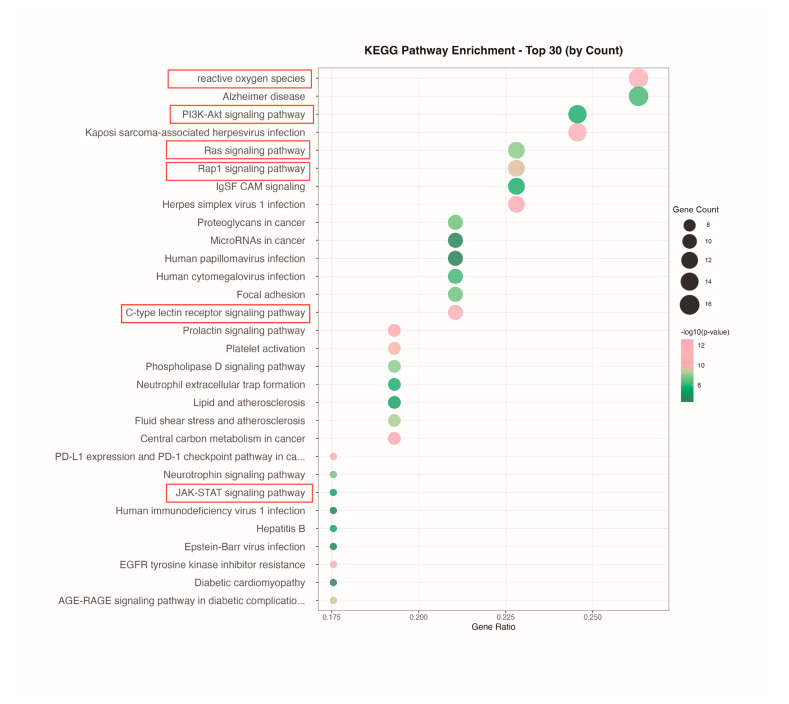
KEGG pathway enrichment analysis. The top 30 pathways ranked by gene count are shown.

KEGG pathway analysis identified 136 enriched pathways, with the top 30 displayed by gene count (Figure 5). Enrichment of the PI3K-Akt signaling pathway indicates PET-MPs disrupt hepatocyte survival, metabolism, and proliferation. Co-enrichment of Ras and Rap1 pathways demonstrates perturbation of growth factor signaling and cell adhesion, contributing to intrahepatic inflammation and fibrosis. Upregulation of ROS pathways suggests oxidative stress as a primary injury mechanism, causing lipid peroxidation and mitochondrial dysfunction while amplifying metabolic inflammation via AGE-RAGE and JAK-STAT pathways. Activation of immune-inflammatory pathways, including platelet activation, neutrophil extracellular trap formation, and C-type lectin receptor signaling, reflects excessive innate immune responses. These interconnected pathways constitute a hepatotoxic network linking metabolic dysregulation, oxidative damage, and immune-inflammatory cascades.

### 2.5. Molecular Docking

**Figure 6:**
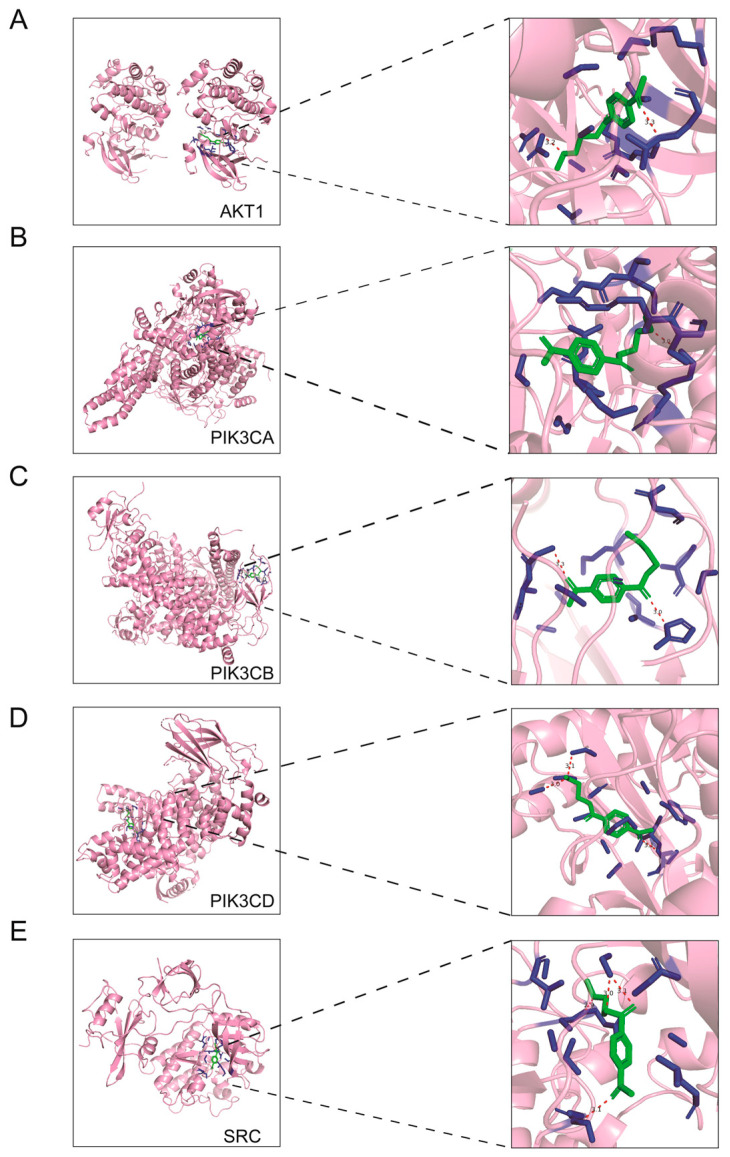
Molecular docking analysis of PET with core targets. Binding mode and affinity between PET and (**A**) AKT1, (**B**) PIK3CA, (**C**) PIK3CB, (**D**) PIK3CD and (**E**) SRC were evaluated using the CB-Dock2 platform.

To validate the predicted interactions between PET and core target proteins, molecular docking simulations were performed. Three-dimensional structures of hub proteins (AKT1, PIK3CA, PIK3CB, PIK3CD, and SRC) were obtained from the PDB, and binding modes and affinities were evaluated using the CB-Dock2 platform, with visualization of the docking results conducted using PyMOL (Figure 6A–E).

Molecular docking results showed that PET binds to all five core target proteins with stable binding affinities, with binding free energies ranging from −6.1 to −7.4 kcal/mol (AKT1: −6.8 kcal/mol; PIK3CA: −7.1 kcal/mol; PIK3CB: −7.4 kcal/mol; PIK3CD: −6.1 kcal/mol; SRC: −6.3 kcal/mol) (Table 2). According to the established criteria, binding free energies <−5 kcal/mol indicate a spontaneous binding interaction, while values <−7 kcal/mol reflect strong and stable binding. Among these, PIK3CA and PIK3CB exhibited the highest binding affinities with PET, suggesting they may be key targets mediating the hepatotoxic effects of PET-MPs. The stable binding between PET and these core proteins indicates a direct interaction, which may interfere with the normal structure and function of the target proteins, thereby disrupting the PI3K-AKT signaling pathway and contributing to liver injury.

### 2.6. Identification and Characterization of PET

**Figure 7:**
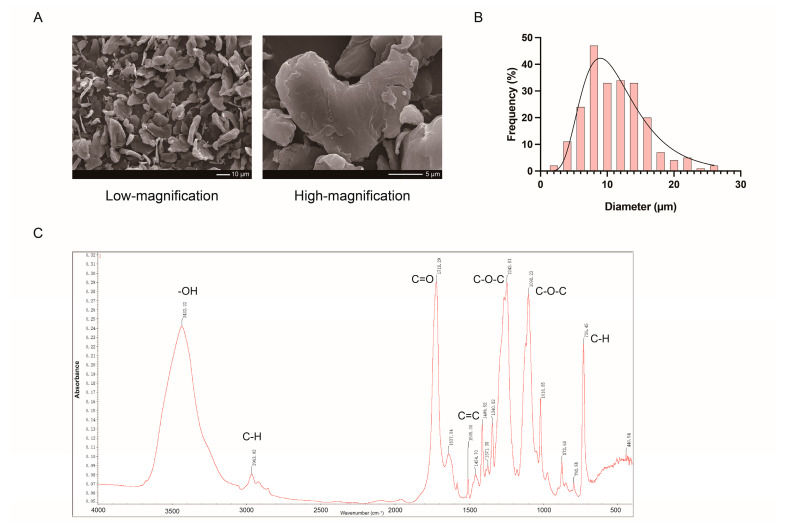
Characterization of PET-MPs. (**A**) SEM images of PET-MPs at low and high magnification, revealing irregular flaky morphology and rough surface. Scale bars: 10 µm (low magnification) and 5 µm (high magnification). (**B**) Particle size distribution of PET-MPs (2.405–26.934 μm). (**C**) FTIR spectrum of PET-MPs, confirming characteristic peaks of -OH (~3400 cm^−1^), C-H (~2900 and ~720 cm^−1^), C=O (~1720 cm^−1^), C=C (~1600 cm^−1^), and C-O-C (~1240 and ~1090 cm^−1^).

As presented in the corresponding SEM images (Figure 7A), the obtained PET particles exhibited heterogeneous lamellar morphology with a relatively smooth surface, and no obvious cracks or pores were detected under high magnification. Statistical analysis of particle size further uncovered a log-normal distribution profile, with diameters ranging from 2.405 to 26.934 μm (Figure 7B). Notably, ATR-FTIR spectroscopy (Figure 7C) displayed representative absorption bands at 3432.22, 1718.29, 1242.81, 1098.23, and 726.45 cm^−1^. These distinctive spectral features collectively validated the chemical identity of polyethylene terephthalate.

### 2.7. PET Microplastics Induce Apoptosis and Inflammatory Responses via Regulation of the AKT Signaling Pathway in AML12 Cells

**Figure 8:**
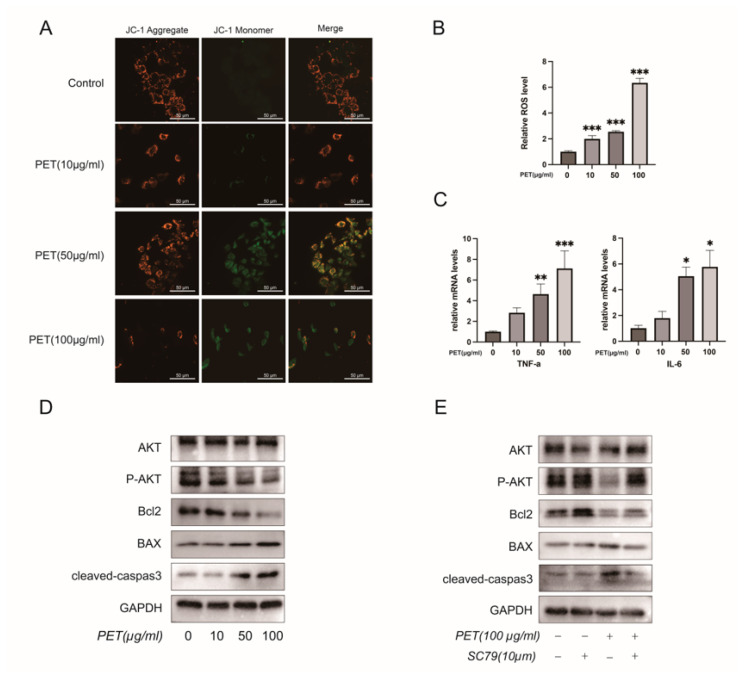
PET-MPs induce mitochondrial dysfunction, oxidative stress, inflammation and apoptosis via inhibiting AKT signaling. (**A**) JC-1 staining shows mitochondrial membrane potential changes after PET-MP treatment. Scale bar: 50 μm. (**B**) Relative intracellular ROS levels. (**C**) mRNA expression of TNF-α and IL-6. (**D**) Western blot analysis of AKT, p-AKT and apoptosis-related proteins. (**E**) Effect of AKT agonist SC79 on PET-MP-induced apoptosis. * *p* < 0.05, ** *p* < 0.01, *** *p* < 0.001 vs. control.

To evaluate the biological effects of PET-MPs on cells, a series of in vitro assays were conducted in this study. JC-1 staining revealed that mitochondrial membrane potential gradually decreased and that green fluorescence intensified with increasing PET concentrations, indicating mitochondrial depolarization and damage (Figure 8A). Accordingly, intracellular ROS levels were gradually elevated as PET concentration increased (Figure 8B). RT-qPCR results demonstrated upregulated mRNA expression of TNF-α and IL-6 (Figure 8C). Western blot further confirmed that PET upregulated apoptotic protein expression and reduced the p-AKT/AKT ratio, thereby promoting cell apoptosis (Figure 8D). After intervention with the AKT agonist SC79, AKT phosphorylation was restored and apoptosis was inhibited, suggesting that activation of the AKT signaling pathway can reverse PET-induced cell apoptosis (Figure 8E).

### 2.8. Activation of the PI3K/AKT Pathway Alleviates PET-MP-Induced Liver Injury In Vivo

**Figure 9:**
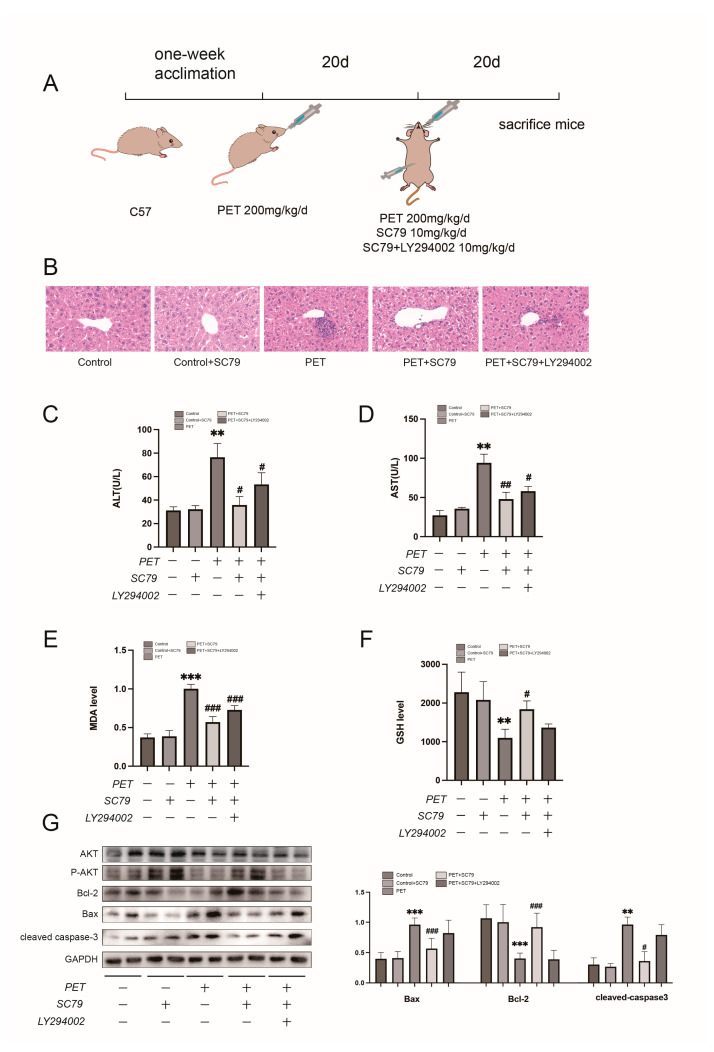
Experimental validation of the PI3K/AKT pathway in PET-induced liver injury. (**A**) Schematic diagram of the experimental design. (**B**) HE staining shows that AKT agonist SC79 alleviates inflammatory cell infiltration. (**C**,**D**) PET significantly increases ALT and AST levels, which are reduced by SC79; this effect is attenuated by LY294002. (**E**,**F**) Measurements of (**E**) MDA and (**F**) GSH levels. (**G**) Western blot analysis confirms that SC79 inhibits PET-induced pro-apoptotic signaling, an effect weakened by LY294002 treatment. Scale bar = 100 μm. Data are represented as the means ± SD. ** *p* < 0.01, *** *p* < 0.001 vs. Control; # *p* < 0.05, ## *p* < 0.01, ### *p* < 0.001 vs. PET.

Based on the network toxicology and molecular docking results, we hypothesized that the PI3K/AKT signaling pathway plays a central role in PET-MP-induced liver injury. To test this hypothesis, we established a mouse model of PET-MP exposure and evaluated the pathway using pharmacological interventions with the AKT agonist SC79 and the AKT inhibitor LY294002. Liver injury was assessed by histopathology, serum liver enzymes, oxidative stress markers, and apoptotic protein expression (Figure 9A).

Histopathological examination revealed significant inflammatory cell infiltration in the portal areas of PET-MP-treated mice (Figure 9B). SC79 treatment markedly reduced this infiltration, and co-treatment with LY294002 reversed the protective effect of SC79. Serum ALT and AST levels were significantly elevated in PET-MP-treated mice (Figure 9C,D). SC79 treatment reduced ALT and AST levels, and this effect was blocked by LY294002.

Biochemical analysis showed that SC79 reduced hepatic MDA content and increased GSH levels in PET-MP-treated mice, indicating reduced oxidative stress (Figure 9E,F). LY294002 treatment attenuated the antioxidant effects of SC79. Western blot analysis showed that SC79 suppressed the PET-MP-induced expression of Bax and Cleaved-Caspase-3 (Figure 9G). LY294002 treatment attenuated the anti-apoptotic effect of SC79.

These results confirm that activating the AKT pathway can effectively alleviate PET-MP-induced liver injury by reducing inflammatory infiltration, improving liver function, inhibiting hepatocyte apoptosis, and regulating the oxidative stress response. This protective effect is dependent on the specific activation of the AKT pathway.

## 3. Discussion

This study focuses on the molecular mechanisms underlying liver injury induced by PET-MPs. Using a systematic research strategy that integrates multi-database target screening, protein interaction network analysis, and in vivo experimental validation, we confirmed the central regulatory role of the PI3K/AKT signaling pathway in PET-MP-induced liver injury.

To identify key molecular targets involved in this process, we first performed cross-target screening using the SwissTargetPrediction and SuperPred databases, identifying 59 potential targets. A PPI network was then constructed via the STRING database and analyzed with Cytoscape. Topological parameters, including degree and betweenness centrality, identified six hub genes (AKT1, PIK3CA, PIK3CB, PIK3CD, PIK3R1, and SRC) that exhibited significantly higher connectivity and centrality within the network. Functional annotation and GO/KEGG enrichment analyses pointed to the PI3K/AKT signaling pathway as a core hub involved in processes such as cell survival, apoptosis, inflammation, and metabolic homeostasis. Based on these findings, we proposed that the PI3K/AKT pathway serves as the central signaling hub mediating PET-MP-induced liver injury.

To test this hypothesis, we first carried out in vitro mechanistic studies using AML12 mouse hepatocytes. PET-MPs induced mitochondrial membrane potential loss and increased reactive oxygen species levels in a concentration-dependent manner, along with upregulating the mRNA expression of the pro-inflammatory factors TNF-α and IL-6. Western blot analysis further confirmed that PET-MPs increased the expression of apoptosis-related proteins while reducing the p-AKT/AKT ratio, indicating inhibition of AKT signaling. Following treatment with the AKT agonist SC79, AKT phosphorylation was restored and apoptosis was reversed, indicating that activating the AKT signaling pathway effectively counteracts PET-MP-induced hepatocyte injury.

We further validated these findings in vivo using a mouse model of PET-MP exposure, with rescue experiments employing the AKT agonist SC79 and inhibitor LY294002. The results showed that SC79-mediated AKT activation effectively alleviated PET-MP-induced liver injury, as evidenced by lower transaminase levels, improved liver histology, and reduced inflammation and oxidative stress. In contrast, LY294002 exacerbated these toxic effects. These results confirm that the protective effect of AKT activation is pathway-specific, bridging the gap between in silico network analysis and in vivo experimental validation.

AKT1, a key effector kinase in the PI3K/AKT pathway, plays core roles in liver physiology and pathology, consistent with its high degree of connectivity in the PPI network [27,28]. Under physiological conditions, AKT1 suppresses hepatocyte apoptosis by phosphorylating downstream targets such as GSK3β and BAD, and also regulates glycogen synthesis and lipid metabolism to maintain hepatic metabolic homeostasis [29]. Our in vitro and in vivo experiments demonstrate that PET-MP exposure disrupts AKT1 activity, leading to reduced anti-apoptotic capacity and aggravated liver injury. This aligns with previous studies linking disrupted AKT1 activity to liver damage induced by environmental pollutants [30,31]. Notably, the rescue effect of SC79 further suggests that restoring AKT1 activity can reverse PET-MP-induced hepatotoxicity, pointing to AKT1 as a potential therapeutic target.

PIK3CA, PIK3CB, and PIK3CD encode the p110α, p110β, and p110δ catalytic subunits of PI3K, respectively [32]. Together, they form the initiation unit for PI3K/AKT pathway activation and display distinct tissue distribution and functional specificity in the liver [33]. The expressed p110α (encoded by PIK3CA) plays a dominant role in maintaining hepatocyte survival and metabolic homeostasis [34]. Our network analysis suggests that PET-MPs may downregulate PIK3CA expression, thereby inhibiting PI3K activation and reducing AKT-mediated anti-apoptotic signaling, consistent with the decreased p-AKT/AKT ratio observed in vitro. PIK3CB (p110β) is involved in maintaining hepatic insulin sensitivity and glucose homeostasis [35]. Its dysregulation by PET-MPs may contribute to metabolic disturbances such as abnormal hepatic lipid accumulation seen in vivo. PIK3CD (p110δ) is mainly expressed in immune cells and regulates liver inflammation [36,37]. Given the elevated pro-inflammatory factors in our mouse model and the concentration-dependent upregulation of TNF-α and IL-6 mRNA in vitro, PET-MPs promote inflammatory activation by upregulating PIK3CD. Collectively, these findings suggest that PET-MPs may promote liver injury through multiple pathways, including hepatocyte damage, fibrosis progression, and inflammatory activation, by regulating these PI3K catalytic subunits.

PIK3R1 encodes the p85α regulatory subunit of PI3K, a core negative regulator of the PI3K/AKT pathway [38]. Under basal conditions, p85α maintains low pathway activity by binding to the catalytic subunit. Upon stimulation, it undergoes conformational rearrangement to release inhibition [39]. Our results suggest that PET-MP exposure may disrupt PIK3R1 expression or modification, leading to aberrant AKT pathway activity. This mechanism is consistent with previous studies showing that dysfunction of PIK3R1 contributes to liver injury by disrupting PI3K/AKT signaling [38,40,41].

SRC, a non-receptor tyrosine kinase, is an important upstream regulator of the PI3K/AKT pathway [42] and a key hub gene in our PPI network. SRC indirectly activates PI3K/AKT by phosphorylating growth factor receptors (e.g., EGFR) [43] or signaling adaptor proteins (e.g., IRS1) [44], and is involved in crosstalk with pathways such as Ras and NF-κB [45,46]. All of which were enriched in our KEGG analysis as contributors to PET-MP-induced liver injury. SRC is a crucial regulator of integrins, clathrin, and dynamin, playing key roles in phagocytosis and macropinocytosis [47,48]. Studies have shown that SRC activation promotes clathrin-mediated endocytosis and phagosome maturation, thereby mediating cellular uptake of micron-sized particles [49,50]. In line with previous reports, the cellular uptake and subsequent biological effects of PET-MPs are highly size dependent [51,52,53]. Smaller PET-MPs can be efficiently internalized by hepatocytes via multiple endocytic pathways, including clathrin-mediated endocytosis, phagocytosis, and micropinocytosis [54,55]. SRC may act as a central regulator governing these internalization processes. SRC modulates the activity of integrins, clathrin, and dynamin, which are essential for membrane invagination, vesicle formation, and particle engulfment [48,56,57]. Activation of SRC has been directly shown to promote clathrin-mediated endocytosis and phagosome maturation, thereby facilitating the uptake of micron-sized particles [58,59]. By contrast, larger MPs are less likely to be fully internalized and may instead trigger outside-in mechanochemical signaling through SRC-dependent integrin phosphorylation [53,57]. Taken together, these findings confirm that SRC serves as a key molecular switch that mediates size-dependent cell entry and signaling of PET-MPs. Therefore, SRC may serve as a critical initiating event in PET-MPs hepatotoxicity by mediating size-dependent cell–particle interactions, contributing to hepatic injury and inflammatory responses. This is consistent with previous studies showing that SRC promotes organ injury induced by environmental pollutants [60,61], further supporting SRC’s role as an upstream regulator of PI3K/AKT signaling in PET-MP hepatotoxicity.

Importantly, these hub genes do not act in isolation, but form a collaborative regulatory network that collectively maintains hepatic cellular homeostasis. SRC, as an upstream activator, can relieve the inhibition of PI3K catalytic subunits by phosphorylating tyrosine residues on PIK3R1, thereby activating the PI3K/AKT signaling axis [62]. AKT1 regulates cell survival and metabolism by phosphorylating downstream substrates and forms a dynamically balanced regulatory loop through negative feedback mechanisms involving crosstalk with SRC and interaction with PIK3R1 [63,64]. This intertwined positive and negative feedback network enables precise tuning of PI3K/AKT signaling in response to stimuli [65]. PET-MPs may disrupt this intrinsic balance. SRC overactivation could lead to sustained phosphorylation of PIK3R1, attenuating its inhibition of catalytic subunits and causing aberrant PI3K/AKT hyperactivation that promotes hepatic stellate cell activation and fibrosis; on the other hand, an imbalance in AKT1 feedback regulation may increase hepatocyte sensitivity to apoptotic stimuli, exacerbating liver cell damage [64]. Thus, the disruption of the hub gene network induced by PET-MPs may represent a core molecular event in their hepatotoxicity.

Beyond the PI3K/AKT pathway, GO and KEGG enrichment analyses suggest that PET-MPs may interfere with normal liver functions through multiple pathways, including Ras, insulin resistance, HIF-1, C-type lectin receptor signaling, apoptosis, and VEGF. These pathways collectively trigger a range of toxic effects—hepatocyte apoptosis, oxidative stress, inflammation, and metabolic disturbances—that are highly consistent with our in vitro and in vivo findings and align with existing preclinical studies [66,67,68,69,70]. Notably, by identifying the PI3K/AKT pathway as a central hub integrating these multiple toxicity pathways, this study extends previous findings and provides a unified mechanistic framework for PET-MP-induced liver injury.

This study adopts a systems biology perspective, viewing biological systems as interconnected complex networks to comprehensively assess the impact of environmental PET-MP exposure, thereby revealing the potential toxicological mechanisms underlying PET-MP-induced liver injury. However, several limitations should be addressed in future research. First, the data used to construct the toxicological network are derived from diverse sources with varying quality, which may affect the accuracy of the analysis. Future studies should validate potential targets using additional databases or experimental methods (such as RNA sequencing) to improve reliability. Second, while molecular docking confirmed favorable binding affinities between PET and the five core targets, detailed binding mode analysis was not performed; future studies employing molecular dynamics simulations or biophysical assays are needed to validate precise binding interactions. Third, this study used a static animal model focusing on short-term PET-MP exposure with mixed particle sizes, which may not fully reflect long-term, low-dose, real-world exposure scenarios or size-dependent effects. Future studies should incorporate size-fractionated PET-MPs and multi-omics approaches to simulate real-world exposure scenarios. Furthermore, other pathways identified in the enrichment analysis (such as NF-κB, HIF-1) may also play important roles, and their crosstalk with the PI3K/AKT pathway warrants further exploration.

## 4. Materials and Methods

### 4.1. Prediction of PET Toxicity

The chemical structure of PET and its corresponding simplified molecular input line entry system (SMILES) notation were retrieved from the PubChem database (https://pubchem.ncbi.nlm.nih.gov/, accessed on 8 January 2025) using “polyethylene terephthalate” (PubChem CID: 18721140) as the keyword. The SMILES string of the PET repeating unit used in this study was CC(=O)C1=CC=C(C=C1)C(=O)OCCOC, which was subsequently imported into ProTox-3.0 (https://tox-new.charite.de/protox3/, accessed on 8 January 2025) and ADMETlab 3.0 (https://admetlab3.scbdd.com/, accessed on 8 January 2025) two web-based platforms for toxicity prediction. After inputting the structure, the corresponding toxicity prediction results were separately downloaded from each platform.

### 4.2. Prediction of PET Target Genes

SwissTargetPrediction (http://www.swisstargetprediction.ch/, accessed on 9 January 2025) and SuperPred 3.0 (https://prediction.charite.de/, accessed on 9 January 2025) were used to predict potential protein targets based on the SMILES information of PET. The target genes obtained from the two databases were merged to form a union set (excluding duplicate genes), and the R package ggvenn (Version 0.1.10) was used to visualize the unique and overlapping targets of the two databases, with the final union set defined as the putative PET-MPs target genes for subsequent analysis.

### 4.3. Identification of Liver Injury-Related Genes

To systematically compile genes associated with liver injury, this study integrated data from three major public databases: GeneCards (https://www.genecards.org/, accessed on 10 January 2025), the Online Mendelian Inheritance in Man (OMIM, https://omim.org/, accessed on 10 January 2025), and the Therapeutic Target Database (TTD, https://db.idrblab.net/ttd/, accessed on 10 January 2025). Candidate genes were retrieved using the keywords “liver injury”. For the GeneCards database, genes with a relevance score greater than 10 were selected to filter for more significant associations. The union of results from all three databases was then extracted to create a set of liver injury-related genes supported by multiple sources.

### 4.4. Identification of Common Target Genes and Enrichment Analysis

The potential target genes of PET-MPs were intersected with the liver injury-related gene set to obtain the common genes, which are considered potential targets of PET-induced liver injury. Functional annotation and pathway enrichment analyses were performed on these common genes. Gene ontology (GO) functional analysis, covering biological processes (BPs), cellular components (CCs), and molecular functions (MFs), and Kyoto Encyclopedia of Genes and Genomes (KEGG) pathway analysis were conducted using R packages (version 4.2.1), including “clusterProfiler” (version 4.4.4), “org.Hs.eg.db” (version 3.15.0), and “enrichplot” (version 1.16.2). Enrichment results with a *p*-value < 0.05 and a false discovery rate (FDR) < 0.05 were considered statistically significant. The results were visualized using R packages such as “ggplot2” (version 3.3.6) and “circlize” (version 0.4.15) to display significantly enriched GO terms and KEGG pathways.

### 4.5. Protein–Protein Interaction (PPI) Network Analysis and Hub Gene Identification

The intersecting genes were imported into the Search Tool for the Retrieval of Interacting Genes (STRING) database (https://string-db.org/, version 11.5) to acquire PPI information. The analysis was confined to Homo sapiens, and a minimum required interaction score of 0.7 (high confidence) was set as the threshold. The resulting interaction data were exported and visualized using Cytoscape software (version 3.9.1). Within this network, topological parameters were analyzed using the NetworkAnalyzer tool (version 4.5.0). The node degree, which represents the number of interactions a given protein has, was used as the primary evaluation metric. Genes ranked in the top 15% by degree value were screened as hub genes.

### 4.6. Molecular Docking Validation

To validate the binding affinity between PET and the hub genes, molecular docking was performed. The three-dimensional crystal structures of the proteins corresponding to the hub genes were obtained from the RCSB Protein Data Bank (PDB, https://www.rcsb.org/, accessed on 11 November 2025), including PIK3CD (PDB ID: 2WXR), AKT1 (PDB ID: 3OCB), PIK3CA (PDB ID: 4L2Y), PIK3CB (PDB ID: 2Y3A), and SRC (PDB ID: 2SRC). The 3D chemical structure of the PET monomer was retrieved from the PubChem database in SDF format. Both structures (protein receptors and PET ligand) were submitted to the CB-Dock2 online docking platform (https://cadd.labshare.cn/cb-dock2/, accessed on 11 November 2025), which performs blind docking by predicting binding sites and calculating binding affinities. All valid docking complexes were visualized and graphically rendered using PyMOL v2.x. The docking results, including binding poses and affinity scores (kcal/mol), were directly obtained from the CB-Dock2 output. Binding energies <−5 kcal/mol were considered indicative of a meaningful binding affinity, and binding energies <−7 kcal/mol were defined as representing a stable binding interaction.

### 4.7. Reagents

PET-MPs were purchased from Shanghai Yuanfang Industrial Co., Ltd. (Shanghai, China). The SEM observation and FTIR detection of PET were conducted at Hunan Kewei Testing Technology Co., Ltd. (Changsha, Hunan, China) to characterize its surface morphology and chemical structure. Protein lysis buffer was obtained from ComWin Biotech Co., Ltd. (Beijing, China). Protease inhibitor cocktail (#SL1086) and phosphatase inhibitor cocktail (#SL1087) were purchased from Coolaber (Beijing, China). ALT (#C009-2-1) and AST (#C010-2-1) assay kits were acquired from Nanjing Jiancheng Bioengineering Institute (Nanjing, Jiangsu, China). JC-1 (#KTA4001), malondialdehyde (MDA) (#KTB1050), glutathione (GSH) (#KTB1600) assay kits and ECL chemiluminescence substrate (#BMU102) were sourced from Abbkine (Wuhan, Hubei, China). Antibodies against Bax (#R380709), Bcl-2 (#381702), and Cleaved-Caspase-3 (#3341034) were obtained from Zenbio (Chengdu, Sichuan, China). Antibodies against ACTIN (#PTR2364), P-AKT (#PT0470R) and AKT (#PT0654R) were purchased from Immunoway (Plano, TX, USA). GAPDH (#AB-P-R001) antibody was purchased from Goodhere (Hangzhou, Zhejiang, China) and used at a dilution of 1:800. All other primary antibodies (Bax, Bcl2, Cleaved-Caspase-3, P-AKT, AKT, ACTIN) were used at a dilution of 1:1000. SC79 (HY-18749) and LY294002 (HY-10108) were purchased from MedChemExpress (Monmouth Junction, NJ, USA).

### 4.8. Cell Culture

The mouse hepatocyte cell line AML12 was purchased from the Cell Bank of the Chinese Academy of Sciences. Cells were cultured in high-glucose Dulbecco’s Modified Eagle Medium (DMEM) supplemented with 10% fetal bovine serum (FBS) and 1% penicillin–streptomycin (100 U/mL penicillin, 100 μg/mL streptomycin) in a humidified incubator at 37 °C with 5% CO_2_. The culture medium was replaced every 24–48 h, and cells were passaged when they reached 80–90% confluence using 0.25% trypsin–EDTA for digestion.

### 4.9. Detection of Intracellular ROS Level

Intracellular reactive oxygen species (ROS) were detected using 2′,7′-dichlorodihydrofluorescein diacetate (DCFH-DA). After the cells were treated with PET microplastics, the culture medium was discarded, and the cells were incubated with DCFH-DA working solution (10 μmol/L) at 37 °C for 20 min in the dark. After washing with PBS three times to remove unbound probes, the fluorescence intensity was measured by a fluorescence microplate reader at an excitation wavelength of 488 nm and an emission wavelength of 525 nm, so as to reflect the intracellular ROS level.

### 4.10. JC-1

The mitochondrial membrane potential was detected by JC-1 staining. AML12 cells were seeded in 6-well plates and treated with 0, 10, 50, 100 μg/mL PET for 72 h. After treatment, cells were washed twice with pre-cooled PBS, incubated with 1 mL JC-1 working solution at 37 °C in 5% CO_2_ for 20 min in the dark, then washed twice with JC-1 buffer. Fluorescence was observed under a fluorescence microscope, where red fluorescence indicates normal membrane potential and green fluorescence indicates depolarization, with relative intensity analyzed to assess mitochondrial damage.

### 4.11. Animal Experiment Design

To validate the in vivo hepatotoxicity of PET-MPs and the role of the PI3K/AKT signaling pathway, a mouse model of PET-MP exposure was established, followed by pharmacological intervention using an agonist and an inhibitor.

#### 4.11.1. Experimental Animals and Grouping

Twenty-five healthy 6-week-old male C57BL/6J mice were randomly divided into the following five groups:

Control group: Gavage with an equal volume of saline.

SC79 control group: Based on saline gavage, intraperitoneal injection of the AKT agonist SC79 (10 mg/kg/day).

PET model group: Gavage with PET-MP suspension (200 mg/kg/day).

PET + SC79 treatment group: Based on PET-MP gavage, intraperitoneal injection of the AKT agonist SC79 (10 mg/kg/day).

PET + SC79 + LY294002 rescue group: Based on PET-MP and SC79 treatment, intraperitoneal injection of the AKT inhibitor LY294002 (10 mg/kg/day).

All interventions lasted for 40 days.

#### 4.11.2. Sample Collection and Processing

At the end of the experiment, blood and liver tissues were collected from the mice. Blood samples were centrifuged to obtain serum, which was stored at −80 °C for liver function index analysis. Part of the liver tissue was fixed in 4% paraformaldehyde for paraffin embedding and subsequent pathological analysis. The remaining liver tissue was rapidly frozen in liquid nitrogen and transferred to a −80 °C freezer for Western blot and oxidative stress indicator detection.

#### 4.11.3. Liver Function and Oxidative Stress Indicator Detection

The activities of serum alanine aminotransferase (ALT) and aspartate aminotransferase (AST) were measured using an automatic biochemical analyzer to assess the degree of hepatocyte injury.

Corresponding assay kits were used according to the manufacturer’s instructions to detect the content of malondialdehyde (MDA) and glutathione (GSH) in liver tissue homogenates, respectively, to evaluate the level of oxidative stress.

#### 4.11.4. Histopathological Analysis

Fixed liver tissues were embedded in paraffin, sectioned (4–5 μm thickness), and stained with hematoxylin and eosin (H&E). Pathological changes, including inflammatory cell infiltration, hepatocyte degeneration and necrosis, and portal area lesions, were carefully examined.

### 4.12. Western Blot Analysis

Total protein was extracted from liver tissues, and protein concentration was determined using the BCA method. After electrophoresis, membrane transfer, and blocking, the membranes were incubated with specific primary antibodies (against AKT, p-AKT, Bax, Cleaved-Caspase-3, the internal reference GAPDH, and ACTIN) overnight at 4 °C. Subsequently, the membranes were incubated with corresponding horseradish peroxidase-conjugated secondary antibodies at room temperature, followed by development using ECL chemiluminescent reagent. The grayscale values of the target bands were analyzed using ImageJ software (version 1.54g), and semi-quantitative analysis was performed based on the ratio of the target protein to the internal reference protein.

### 4.13. RT-PCR

Total RNA was extracted from cells or tissues using Trizol reagent according to the manufacturer’s instructions. The purity and concentration of RNA were detected by a nucleic acid analyzer. Reverse transcription was performed to synthesize cDNA using a reverse transcription kit. RT-qPCR was carried out on a real-time fluorescent quantitative PCR instrument with β-actin as the internal reference gene. The primer sequences used for RT-qPCR are listed in Supplementary Material S4. The relative expression levels of target genes were calculated using the 2^−^ΔΔCt method.

### 4.14. Statistical Analysis

Data represent the mean ± standard deviation (SD) of three independent biological experiments performed in triplicate. Statistical comparisons were analyzed using GraphPad Prism version 10. One-way ANOVA was used to assess differences among multiple groups. Statistical significance was set at *p* < 0.05.

## 5. Conclusions

This study used network toxicology and experimental validation to investigate the molecular mechanisms of PET microplastic-induced liver injury. In silico analyses focused on the PET repeating unit, while functional experiments were conducted with PET-MPs. Our results show that PET-MPs induce liver injury through the PI3K/AKT signaling pathway. These findings provide insight into the hepatotoxicity of environmental microplastics and may inform health risk assessment and prevention strategies.

## Figures and Tables

**Table 1:** Topological parameters of hub genes identified from the PPI network.

Name	Betweenness	Closeness	Degree
AKT1	0.382	0.534	20
SRC	0.0455	0.464	17
PIK3CA	0.099	0.469	17
PIK3R1	0.054	0.464	16
PIK3CB	0.014	0.448	14
PIK3CD	0.014	0.448	14

**Table 2:** Molecular docking results revealed that PET could spontaneously bind to five core targets (AKT1, PIK3CA, PIK3CB, PIK3CD and SRC) with high affinity.

Molecular	Score	Volume	Center (x, y, z)	Docking Size (x, y, z)
AKT1	−6.8	2202	57, 1, 19	20, 29, 20
PIK3CA	−7.1	1719	−7, −48, −49	20, 20, 20
PIK3CB	−7.4	1525	20, −78, 3	27, 20, 27
PIK3CD	−6.1	1112	−13, −29, 22	34, 20, 20
SRC	−6.3	892	−18, 26, 58	20, 20, 20

## Data Availability

Data supporting the findings of this study are derived from publicly available databases and computational platforms, including PubChem, ProTox-3.0, ADMETlab 3.0, SwissTargetPrediction, SuperPred, GeneCards, OMIM, TTD, STRING, PDB, and CB-Dock2, as detailed in the Section 4. The processed data generated during this study, including target gene sets, enrichment results, and docking outcomes, are included in the article and its Supplementary Materials.

## Supplementary Materials

The following supporting information can be downloaded at: https://www.mdpi.com/article/10.3390/ijms27073256/s1.
