# Supplementary material for: Hepatotoxic Mechanisms of Polyethylene Terephthalate Microplastics Revealed by Network Toxicology, Molecular Docking, and In Vivo Validation
Source: Int J Mol Sci. 2026 Apr 3;27(7):3256. doi: 10.3390/ijms27073256 (PMC13073197; doi:10.3390/ijms27073256)
Supplement: Supplementary file 1 [file ijms-27-03256-s001.zip › Supplementary Material S4/Supplementary Material S4.pdf]

Supplementary Table 1

Primer

ACTIN	ATCGCTGCGCTGGTCG
	GAGTCCTTCTGACCCATTCCC
IL-6	AGCCAGAGTCCTTCAGAGAGA
	GCCACTCCTTCTGTGACTCC
TNF-a	GATCGGTCCCCAAAGGGATG
	CCACTTGGTGGTTTGTGAGTG
