# Supplementary figures and images for: Hepatotoxic Mechanisms of Polyethylene Terephthalate Microplastics Revealed by Network Toxicology, Molecular Docking, and In Vivo Validation
Source: Int J Mol Sci. 2026 Apr 3;27(7):3256. doi: 10.3390/ijms27073256 (PMC13073197; doi:10.3390/ijms27073256)

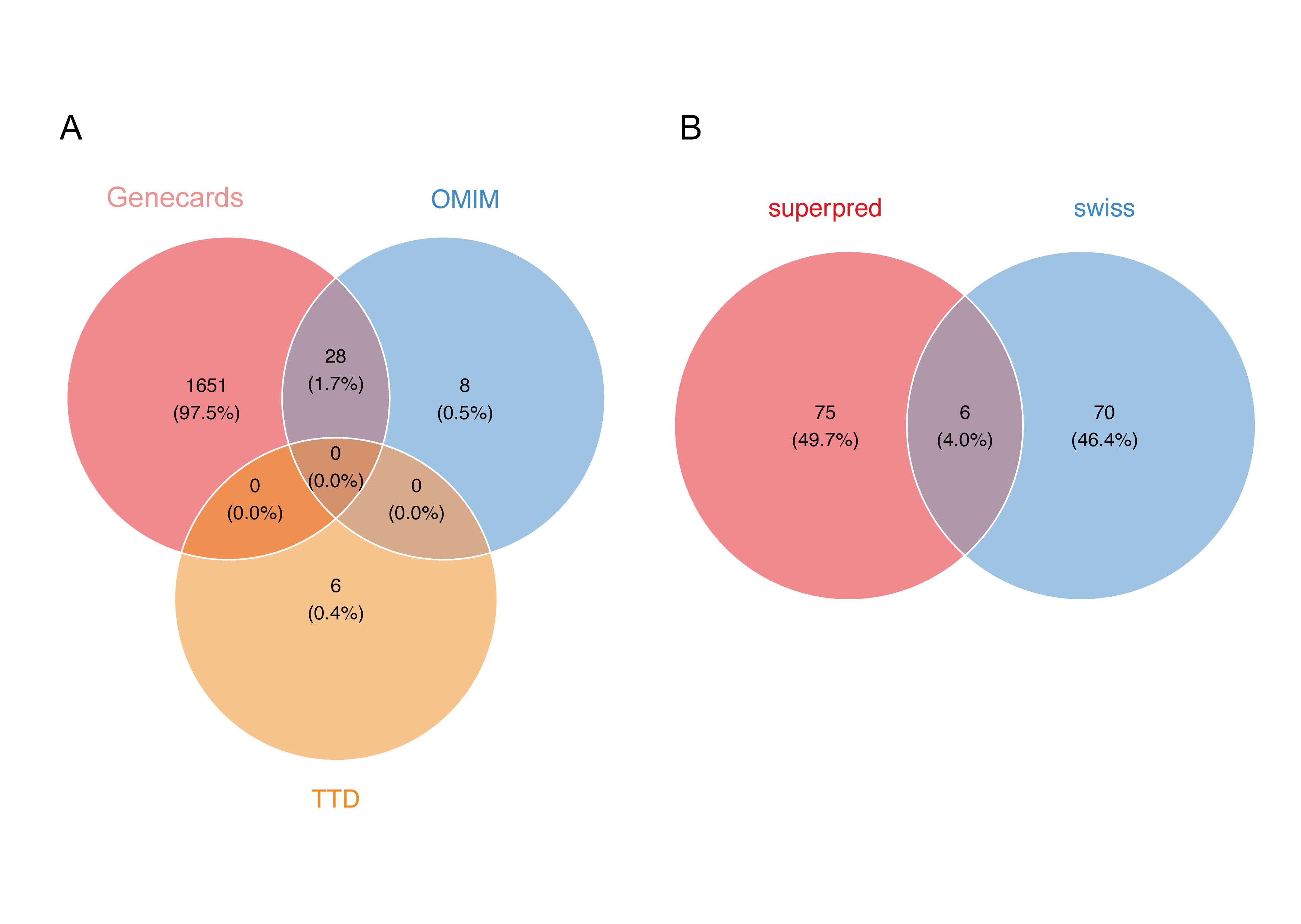

Supplement: Supplementary file 1 [file ijms-27-03256-s001.zip › Supplementary Material S1/Supplementary Material S1.tif]
